# A New Model to Predict Survival Time in Patients With Hepatocellular Carcinoma With BCLC Advanced Stage

**DOI:** 10.1002/cam4.71299

**Published:** 2025-10-15

**Authors:** Masashi Ninomiya, Mio Tsuruoka, Jun Inoue, Atsushi Hiraoka, Akitoshi Sano, Kosuke Sato, Masazumi Onuki, Satoko Sawahashi, Keishi Ouchi, Kengo Watanabe, Hidekatsu Kuroda, Takayoshi Oikawa, Tamami Abe, Masashi Fujita, Kazumichi Abe, Tomohiro Katsumi, Wataru Sato, Go Igarashi, Chikara Iino, Nobukazu Tanabe, Hiroshi Numao, Hiromasa Ohira, Yoshiyuki Ueno, Atsushi Masamune

**Affiliations:** ^1^ Division of Gastroenterology Tohoku University Hospital Sendai Japan; ^2^ Gastroenterology Center Ehime Prefectural Central Hospital Ehime Japan; ^3^ Division of Gastroenterology and Hepatology, Department of Internal Medicine Iwate Medical University Iwate Japan; ^4^ Department of Gastroenterology, School of Medicine Fukushima Medical University Fukushima Japan; ^5^ Department of Gastroenterology, Faculty of Medicine Yamagata University Yamagata Japan; ^6^ Department of Gastroenterology, Graduate School of Medicine Akita University Akita Japan; ^7^ Department of Gastroenterology, Graduate School of Medicine Hirosaki University Hirosaki Japan; ^8^ Department of Gastroenterology National Hospital Organization Sendai Medical Center Sendai Japan; ^9^ Department of Gastroenterology Aomori Prefectural Central Hospital Aomori Japan

**Keywords:** BCLC advanced stage HCC, overall survival, parametric model, survival predictors

## Abstract

The variety of treatments available makes it difficult to determine whether a treatment would be effective for an individual case in advanced‐stage hepatocellular carcinoma. We aimed to establish a new model of survival prediction in patients to establish standards in the recent and coming multimolecular targeted agents era. This analysis was prepared using a data set of 518 patients diagnosed with hepatocellular carcinoma prior to 2017. Multiple regression analysis showed the size of the largest tumor nodule, the number of nodules, macrovascular invasion, extrahepatic metastasis, total bilirubin, albumin, prothrombin time (PT), α‐fetoprotein, and des‐γ‐carboxyprothrombin (DCP) as independent predictors of survival. A Weibull model had the best fit and, based on these predictors, we established two new predicted survival models with or without incorporating PT and DCP. This model is useful for planning and evaluating the efficacy of recent sequential therapies in the multimolecular targeted agents era.

**Abbreviations**
AFP: α‐fetoprotein
ALBI: albumin‐bilirubin
ALT: alanine aminotransferase
AST: aspartate aminotransferase
BCLC: Barcelona Clinic Liver Cancer
BSC: best supportive care
CT: contrast‐enhanced computed tomography
CTLA4: cytotoxic T‐lymphocyte‐associated protein
DCP: des‐γ‐carboxyprothrombin
EASL: European Association for the Study of the Liver
ECOG: Eastern Cooperative Oncology Group
HAIC: hepatic arterial infusion chemotherapy
HCC: hepatocellular carcinoma
ICI: immune‐checkpoint inhibitor
mALBI: modified ALBI
MRI: magnetic resonance imaging
MTAs: molecular targeted agents
PD‐L1: programmed death 1 ligand
PS: performance status
TACE: transcatheter arterial chemoembolization
TAE: transcatheter arterial embolization
TAI: transcatheter arterial infusion
TKI: tyrosine kinase inhibitor

## 1 Introduction

Hepatocellular carcinoma (HCC) is the sixth most common cancer worldwide and the third leading cause of cancer‐related deaths globally [1]. Multiple staging systems have been proposed for HCC, with the Barcelona Clinic Liver Cancer (BCLC) staging system being most commonly utilized by the American Association for the Study of Liver Disease (AASLD) and the European Association for the Study of the Liver (EASL). The BCLC staging system categorizes HCC into five stages, with advanced stage defined as HCC with portal invasion and/or extrahepatic spread [2]. Before the introduction of tyrosine kinase inhibitors (TKIs) for HCC, the 1‐year survival rate was 25% in the advanced stage [3]. In 2008, sorafenib, one of the TKIs, was approved for the treatment of advanced HCC and significantly improved the median overall survival of 10.7 months in the SHARP trial [4]. From 2009 to 2017, Sorafenib was the primary choice for HCC treatment in the advanced stage. Generally, this period was defined as the one‐TKI era.

Currently, several systemic therapies are being utilized for patients with unresectable HCC, primarily including those in the advanced stage. They broadly fall into two groups: antiangiogenic targeted therapies and immune‐checkpoint inhibitors (ICIs). Antiangiogenic targeted therapies include the TKIs (sorafenib, regorafenib, lenvatinib, and cabozantinib) and monoclonal antiangiogenic antibodies (ramucirumab and bevacizumab). ICIs include inhibitors of programmed death 1 ligand (PD‐L1) (atezolizumab and durvalumab) and cytotoxic T lymphocyte‐associated protein 4 (CTLA4) inhibitors (tremelimumab) [4, 5, 6, 7, 8, 9]. The AASLD guidelines recommend atezolizumab–bevacizumab or durvalumab–tremelimumab combination therapy as the first line in the advanced stage [9]. Atezolizumab–bevacizumab combination therapy showed a median overall survival of 19.2 months and durvalumab–tremelimumab of 16.4 months, which was longer than sorafenib monotherapy in each randomized phase III trial [10, 11]. With the development of chemotherapy, survival is expected to be prolonged in the HCC advanced stage. However, in clinical practice, it is rarely the case that only one chemotherapy is completed. The variety of treatments available makes it difficult to determine whether a treatment is effective for an individual case. This is why we created our prognostic model. If the survival time can be predicted by the treatment in a given period, then the actual treatment today can be evaluated. Following the prediction model for the intermediate stage, we developed a model to predict survival in patients with HCC in the advanced stage based on one‐TKI era [12].

## 2 Methods

### 2.1 Study Design and Participants

In this retrospective cohort study, 518 HCC patients were included from 2007 to 2017 at the four liver centers in Japan (Akita University Hospital, Iwate Medical University Hospital, Tohoku University Hospital and Ehime Prefectural Central Hospital). The HCC stage was evaluated according to the BCLC classification and all the cases diagnosed as the advanced stage were enrolled. Patients with Eastern Cooperative Oncology Group (ECOG) PS of 2 or higher were excluded. The clinical information of patients was extracted from their medical records and they were followed to identify their death status via the medical information sheet from their relative hospitals. The survival time was designated as the time between the diagnosis date of HCC advanced stage and the occurrence of death. The death status was considered a failure event. All therapies were allowed, but no patients underwent liver transplantation.

This study was approved by the institutional review board of Tohoku University Hospital (2021‐1‐377). The study was conducted in accordance with the principles of the Declaration of Helsinki (Fortaleza revision, 2013).

### 2.2 Data Collection

The diagnosis of HCC nodules was characterized by contrast‐enhanced computed tomography (CT) or magnetic resonance imaging (MRI), including the number of tumor nodules, the diameter of the largest nodule, and the vascular invasion. Evaluation of the metastasis was determined by CT and/or positron emission tomography (PET). The following clinical parameters and biochemistry data were included in the table: age, gender, etiology, ECOG performance status, total bilirubin, aspartate aminotransferase (AST), alanine aminotransferase (ALT), albumin, platelets, prothrombin (PT) time, α‐fetoprotein (AFP), des‐γ‐carboxyprothrombin (DCP), tumor size, numbers of nodules, macrovascular invasion, extrahepatic metastasis, albumin‐bilirubin (ALBI) score, the modified ALBI (mALBI) grade, Child‐Pugh score, and treatment naïve or recurrence. The ALBI score was calculated using the formula: linear predictor = (log_10_ (total bilirubin × 17.1) × 0.66) + (albumin × 10 × −0.085), and the cut points of the mALBI grade were as follows: × ≤ −2.60 (grade 1), more than −2.60 to < −2.27 (grade 2a), not less than −2.27 to ≤ −1.39 (grade 2b), and more than −1.39 (grade 3) [13, 14]. Continuous variables are presented as median (interquartile range) and categorical variables as numbers. Survival was calculated as the time from the date of the initial diagnosis as the BCLC advanced stage to death by HCC.

### 2.3 Statistical Analysis and Weibull Distribution Model

Patient survival probability was analyzed using the Kaplan–Meier method. The survival‐related factors were extracted by the Cox proportional hazard regression model based on the factors with multivariate significance (*p* < 0.05) and the clinical relevance that has been previously reported.

The survival model was applied based on the Weibull distribution which the previously reported [12]. We evaluated the appropriateness of this parametric model using a probability plot [15]. The cumulative failure probability was defined as *F* (*x*, *α*, *β*) = 1−exp[−(*x*/*β*)^*α*^]. *x* showed the survival time, *α*, scale parameter and *β*, shape parameter. In this case, the median survival time could be calculated as follows

$$F\left(x,\alpha ,\beta \right)=1-\exp\left[-{\left(x/\beta \right)}^{\alpha }\right]=0.5$$

$$x=\beta \;x\;{\left(-\log0.5\right)}^{1/\alpha }$$

*β* is consisted of the statistical weighing and the risk factors.

$$\begin{array}{cc} \beta \;=\; & \exp\;(\text{intercept}+\text{coefficient}1*\left(\text{factor}1\right)\\ & +\text{coefficient}2*\left(\text{factor}2\right)+\ldots ) \end{array}$$

The risk factors were extracted from the multivariate analysis of the Cox proportional hazard regression model [12]. The intercept and the coefficient of each risk factor were determined by JMP Pro 17.1.0 (SAS Institute, Cary, NA).

## 3 Results

### 3.1 The Characteristics of the Patients in This Cohort Study

**FIGURE 1:**
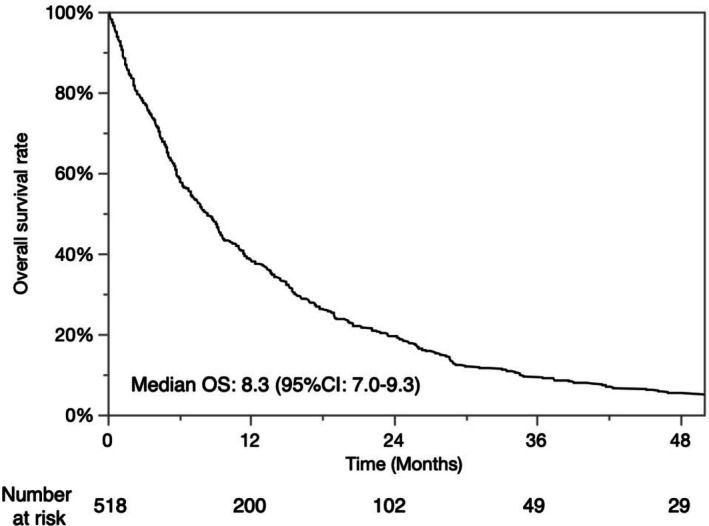
Kaplan–Meier curves for overall survival in this cohort study.

The characteristics of the patients in the cohort are shown in Table 1. Five hundred and eighteen patients were enrolled in this cohort. The median age was 69 years, and the majority were male. Hepatitis C virus was the predominant etiology. There were 484 patients with PS0, while 34 had PS1. There were 189 (36.5%) patients with a Child‐Pugh score of 5, 138 (26.6%) with a score of 6, 85 (16.4%) with a score of 7, and 106 (20.5%) with a score of over 8, corresponding to 327 (63.1%) patients with a Child‐Pugh class A and 191 (36.9%) with up to class B. The median ALBI score was −2.16. There were 119 (23.0%) patients with mALBI grade 1, 104 (20.1%) with grade 2a, 237 (45.7%) with grade 2b, and 58 (11.2%) with grade 3. The median size of the largest nodule was 4.2 cm, and the number of nodules was four. There were 151 (29.2%) HCC patients with major branch of portal/hepatic vein invasion, 212 (42.5%) patients with segmental branch invasion, and extrahepatic spread was shown in 209 (40.3%) cases. The median AFP concentration was 278 ng/dL, and DCP was 1647 mAU/mL. When diagnosed as HCC with the advanced stage, 235 (45.4%) patients were treatment naïve, while 283 (54.6%) were recurrent. As initial treatment at advanced stage, 207 patients were treated with transcatheter arterial (chemo)embolization (TAE/TACE), 101 patients with hepatic arterial infusion chemotherapy (HAIC) or transcatheter arterial infusion (TAI), and 86 patients with some TKI including sorafenib. After progression with the advanced stage, the median overall survival (OS) was 8.3 months by the nonparametric estimator of survival functions (Figure 1).

**TABLE 1:** Description of the patients (*n* = 518).

Patient characteristics	Value	Tumor characteristics	Value
Age, years	69 (62–76)	Macrovascular invasion	
Gender		Major Br. of portal/hepatic veins	151
Male	406	Seg. Br. of portal/hepatic veins	220
Female	112	Without invasion	147
Etiology		Extrahepatic metastasis	
HCV	281	Present	209
HBV	80	None	309
HCV + HBV	9	Size of the largest nodules, cm	4.2 (2.7–7.4)
nonBnonC	148	< 1	17
ECOG, PS 0/1	484/34	1–2	47
Naïve/recurrence	235/283	2–3	78
Total bilirubin, mg/dL	0.9 (0.6–1.5)	3–4	91
AST, U/L	60 (40–97)	4–6	111
ALT, U/L	43 (26–66)	6–10	100
Albumin, g/dL	3.5 (3.1–3.9)	≥ 10	74
Platelets, ×10^4^/μL	13.0 (8.6–18.4)	Number of nodules	4 (2–7)
Prothrombin time, %	88.5 (74.0–97.9)	0	12
AFP, ng/dL	278 (22–5456)	1	96
DCP, mAU/mL	1647 (130–18,956)	2	58
ALBI score	−2.16 (−2.56 to −1.74)	3	44
mALBI grade		4	60
1	119	5	28
2a	104	6	39
2b	237	≥ 7 or diffuse	181
3	58	Initial treatment when advanced HCC is diagnosed	
Child‐Pugh score		Operation	42
5	189	TAE/TACE	207
6	138	HAIC/TAI	101
7	85	Sorafenib	77
≥ 8	106	Chemotherapy	9
		BSC/others	61/21

Abbreviations: AFP, α‐fetoprotein; ALBI, albumin‐bilirubin; ALT, alanine aminotransferase; AST, aspartate aminotransferase; Br, branch; BSC, best supportive care; DCP, des‐γ‐carboxyprothrombin; ECOG, Eastern Cooperative Oncology Group; HAIC, hepatic arterial infusion chemotherapy; HBV, hepatitis B virus; HCV, hepatitis C virus; mALBI, modified ALBI; PS, performance status; Seg, segmental; TACE, transcatheter arterial chemoembolization; TAE, transcatheter arterial embolization; TAI, transcatheter arterial infusion.

### 3.2 Predictor of Survival and Survival Prediction Model

A univariate Cox proportional hazard regression analysis was performed in this cohort (Table 2). The number of nodules, the size of the largest tumor nodule, macrovascular invasion, extrahepatic metastasis, total bilirubin, albumin, platelets, PT time, AFP, and DCP were significantly different. Based on these variables, multiple regression analysis was conducted. The number of nodules, the size of the largest tumor nodule, macrovascular invasion, extrahepatic metastasis, total bilirubin, albumin, PT, AFP, and DCP were selected as independent predictors of survival (Table 2). Since the values showed significant upward deviations, I applied a log scale for better calculation. Specifically, this was done for AFP and DCP.

**TABLE 2:** Univariate and multivariate analyses of derivation cohort by the Cox proportional hazard regression model.

Factors	Category	Univariate		Multivariate	
		Hazard ratio (95% CI)	*p*	Hazard ratio (95% CI)	*p*
Age	Years	1.117 (0.617–2.072)	0.7211		
Gender	Male Female	0.864 (0.700–1.065) 1.158 (0.939–1.428)	0.1705		
Etiology	Viral Nonviral	1.027 (0.849–1.242) 0.974 (0.805–1.177)	0.7837		
Treatment	Naïve Recurrence	0.922 (0.775–1.098) 1.084 (0.911–1.291)	0.3635		
Number of nodules	Variable	1.971 (1.526–2.549)	< 0.0001	1.626 (1.241–2.133)	0.0004
Size of the largest nodule	Variable	3.096 (2.004–4.716)	< 0.0001	1.718 (1.007–2.898)	0.0449
Macrovascular invasion	None/Seg. None/Major Seg./Major	1.077 (0.874–1.328) 0.633 (0.503–0.796) 0.588 (0.476–0.725)	< 0.0001	0.838 (0.623–1.129) 0.674 (0.488–0.930) 0.803 (0.633–1.019)	0.0424
Extrahepatic metastasis	None/Yes	0.791 (0.663–0.943)	0.0091	0.713 (0.552–0.923)	0.0101
Total bilirubin	Variable	23.85 (6.602–62.38)	< 0.0001	10.55 (2.162–33.94)	0.0006
Albumin	Variable	0.145 (0.088–0.241)	< 0.0001	0.288 (0.173–0.481)	< 0.0001
Platelets	Variable	1.457 (0.678–3.010)	0.3291		
Prothrombin time	Variable	0.137 (0.078–0.244)	< 0.0001	0.468 (0.269–0.820)	0.0075
LN(AFP)	Variable	4.050 (2.711–6.043)	< 0.0001	2.183 (1.408–3.391)	0.0005
LN(DCP)	Variable	5.624 (3.431–9.216)	< 0.0001	1.825 (1.007–3.307)	0.0471

*Note:* *p* value < 0.05 denotes statistical significance.

Abbreviations: 95% CI, 95% confidence interval; AFP, α‐fetoprotein; DCP, des‐γ‐carboxyprothrombin.

### 3.3 Development of a New Estimated Survival Model

**FIGURE 2:**
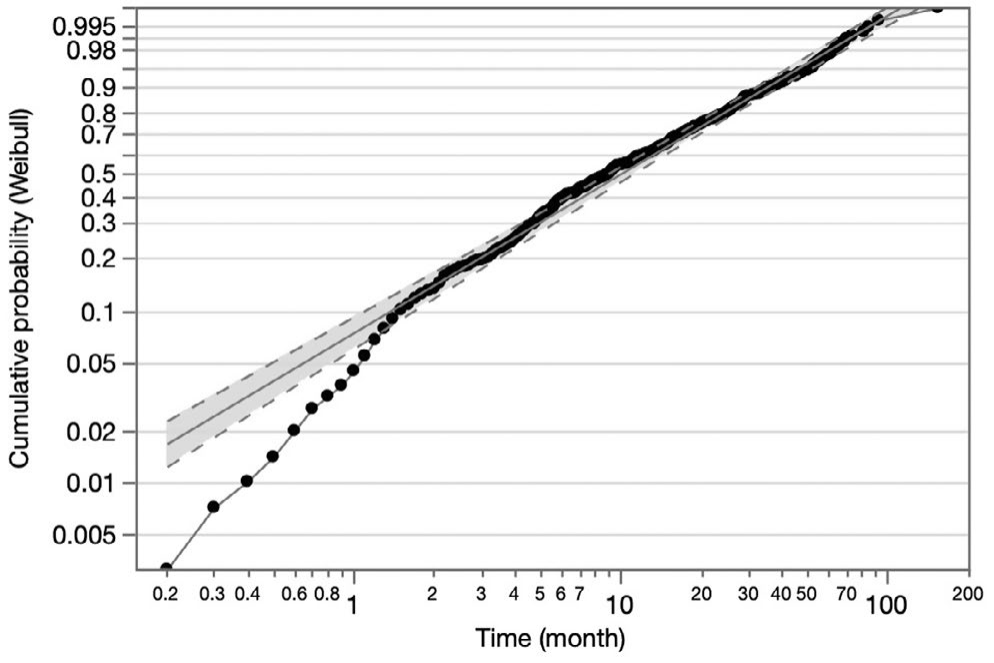
Estimation of parametric model. Probability plots for each distribution. The linear course indicates the graph of log–log survival against the log of failure time in each distribution. The dot‐to‐dot linear shows the survival distribution.

We have already developed a survival model for the intermediate stage of HCC patients, and the Weibull distribution was fitted to predict the survival time [12]. The probability plots can provide a visual check of the appropriateness, and the plots appeared approximately linear (Figure 2). Therefore, we used the Weibull distribution function to develop a new mathematical survival model for the advanced stage of HCC again. Nine predictors were selected by multiple regression analysis and became the candidates for the factors in this model. However, there were some concerns about these predictors. First, we were concerned with how to count the nodules of the numerous or diffuse type accurately. After reviewing the CT or MRI imaging findings with both hepatologists and radiologists, we designated the number of HCC nodules when there were more than 6 or the diffuse type set of 11. Next, PT times and DCP were included as the independent predictors, but we could not obtain accurate values for them when a patient indicated warfarin. We developed two prediction models with and without incorporating PT time and DCP. In the Weibull distribution model, the predicted survival time was derived as [exp (intercept + coefficient1 × (factor1) + coefficient2 × (factor2) + …) × (−log0.5)^1/*α*^]. The nine factors related to the overall survival were selected by Cox proportional hazard regression analysis, which we age‐adjusted. The parameter estimates were calculated by the JMP program shown in Table 3. The 50% survival duration (months) was predicted by exp(2.15824 + (−0.00516897) × age + (−0.074185) × (number of nodules) + (−0.024164) × (the size of the largest nodule, cm) + (−0.20311) × (macrovascular invasion {0 for none/1 for segmental branch/1 for major branch}) + (−0.36639) × (extrahepatic metastasis {0 for none/1 for presence}) + (−0.051600) × (T‐bil, mg/dL) + 0.38771 × (Alb, g/dL) + 0.0089163 × (PT times, %) + (−0.048190) × LN(AFP, ng/dL) + (−0.031375) × LN(DCP, mAU/mL)) × (−LN0.5)^0.93074^ defined as model 1 and exp(2.64253 + (−0.0039024) × age + (−0.080417) × (number of nodules) + (−0.028668) × (the size of the largest nodule, cm) + (−0.21687) × (macrovascular invasion {0 for none/1 for segmental branch/1 for major branch}) + (−0.38303) × (extrahepatic metastasis {0 for none/1 for presence}) + (−0.060143) × (T‐bil, mg/dL) + 0.42834 × (Alb, g/dL) + (−0.059758) × LN(AFP, ng/dL)) × (−LN0.5)^0.93219^ defined as model 2 (Data S1).

**TABLE 3:** The coefficient of each factor in Weibull survival model.

Factors	Input variable	Model 1		Model 2	
		Coefficients	Standard error	Coefficients	Standard error
Intercept		2.15824	0.49644	2.64253	0.43692
Age	Years	−0.00516897	0.00440542	−0.0039024	0.0043521
Number of nodules	Under 6: Numbers Over 6: 7 Diffuse type: 7	−0.074185	0.017976	−0.080417	0.017795
Size of the largest nodule	Size (cm)	−0.024164	0.013266	−0.028668	0.012607
Macrovascular invasion	None: 0 Segmental branch: 1 Major branch: 2	−0.20311	0.077207	−0.21687	0.075644
Extrahepatic metastasis	None:0 Presence: 1	−0.36639	0.10969	−0.38303	0.10632
Total bilirubin	Value (mg/dL)	−0.051600	0.014086	−0.060143	0.013470
Albumin	Value (g/dL)	0.38771	0.076609	0.42834	0.074850
Prothrombin time	Value (%)	0.0089163	0.0033777		
LN(AFP)	Value (ng/dL)	−0.048190	0.014557	−0.059758	0.013948
LN(DCP)	Value (mAU/mL)	−0.031375	0.018931		
1/*α*		0.93074	0.030745	0.93219	0.030895

Abbreviations: AFP, α‐fetoprotein; DCP, des‐γ‐carboxyprothrombin.

## 4 Discussion

In this study, we demonstrated the predictors of survival in advanced‐stage HCC patients and developed a mathematical model to estimate the survival time based on the predictors of the parametric distribution. The Weibull distribution had the best fit among all the investigated parametric models [12]. Using our new parametric survival model, we achieved more flexibility in predicting the survival duration in patients with advanced‐stage HCC. Using this newly developed survival model, predicting survival time assuming that the patient would have lived in the one‐TKI era allows us to evaluate whether current treatment is effective. This model could be recommended for planning, health policymaking, and the evaluation of treatments and, potentially, it may contribute to improving the survival of patients with HCC.

Before 2017, Sorafenib had been shown to be effective in the first line and 10.7 months in overall survival with the advanced stage [4]. Therefore, the EASL guidelines recommend treating advanced‐stage HCC patients with systematic therapy and showed 10 months as the estimated mean survival time [16]. After 2017, several molecular targeted agents (MTAs) and ICIs were approved for HCC therapy with the advanced stage. Nowadays, systemic therapies with atezolizumab plus bevacizumab or durvalumab plus tremelimumab are considered the preferred first line therapy [17]. The median OS of 19.2 months with atezolizumab plus bevacizumab and 16.4 months with durvalumab plus tremelimumab was longer than the treatment with sorafenib. However, the overall response rate by RECIST was still low, showing 20%–30% and could not lead to complete remission with all advanced‐stage HCC patients [10, 11]. Therefore, second line therapy and beyond were important, but all second line clinical trials were conducted after sorafenib in the first line setting, and no high‐quality data have been published on the second line therapy after atezolizumab plus bevacizumab or durvalumab plus tremelimumab. In the AASLD guidelines, sorafenib or lenvatinib are the preferred agents after the first line atezolizumab plus bevacizumab or durvalumab plus tremelimumab. The indication for cabozantinib, regorafenib, or ramucirumab is employed after sorafenib or Lenvatinib [9]. Therefore, in the real‐world, multiple treatments such as a combination and/or sequential therapy are usually administered for HCC patients in the advanced stage. Actually, it is difficult to evaluate the effectiveness of sequential systemic therapies. Given these circumstances, we developed a mathematical model to estimate the survival duration in advanced‐stage HCC patients, and we can now evaluate the efficacy of recent sequential therapy to determine whether it is appropriate or not.

However, there are several limitations. One limitation is that the model has not been validated through a formal validation study. Nevertheless, we consider this prediction model to be a work in progress, which will be continuously updated and validated as more cases are accumulated. Additionally, in cases where ICIs are used at the intermediate stage, their effects may persist even after the disease progresses to the advanced stage. This could lead to inaccuracies in the model, which we recognize as a challenge to be addressed in future research.

We have already developed a mathematical model to estimate the survival time in intermediate‐stage HCC patients. In this trial, we identified six factors such as naïve/recurrence, number of nodules, size of the largest tumor, total bilirubin, albumin, and AFP levels as independent predictors of overall survival and applied them as parameters, including age. While the advanced stage differs from the intermediate stage in terms of PS, there is the presence of vascular invasion and metastasis. In addition to these three components, we reviewed previous papers for validity. Although many prognostic models for the advanced stage have been reported, most of the treatment models are based on sorafenib alone. In addition, since various treatments are given continuously in the advanced HCC stage [18], it is desirable to create a model that reflects overall survival. In 2018, Giannini et al. reported that the survival time in advanced‐stage HCC patients was related to the Model for End‐stage Liver Disease score, Child‐Pugh class, ascites, platelet count, albumin, tumor size, macrovascular invasion, extrahepatic metastasis, and AFP [19]. We also analyzed the predictors of overall survival in our own cohort and nine factors were extracted. These were similar to previous reports. Then, we developed a survival model using these parameters. This model is consistent with all routinely available parameters, and it is simple to calculate using common calculation software. The developed model we have created will be extremely useful for evaluating sequential treatments within the survival time under real‐world clinical practice.

## Author Contributions

**Masashi Ninomiya:** data curation (equal), formal analysis (equal), project administration (equal), writing – original draft (equal). **Mio Tsuruoka:** data curation (equal). **Jun Inoue:** supervision (equal), validation (equal). **Atsushi Hiraoka:** investigation (equal). **Akitoshi Sano:** data curation (equal). **Kosuke Sato:** data curation (equal). **Masazumi Onuki:** data curation (equal). **Satoko Sawahashi:** data curation (equal). **Keishi Ouchi:** data curation (equal). **Kengo Watanabe:** data curation (equal). **Hidekatsu Kuroda:** data curation (equal), investigation (equal). **Takayoshi Oikawa:** data curation (equal). **Tamami Abe:** data curation (equal). **Masashi Fujita:** data curation (equal). **Kazumichi Abe:** data curation (equal). **Tomohiro Katsumi:** data curation (equal). **Wataru Sato:** data curation (equal). **Go Igarashi:** data curation (equal). **Chikara Iino:** data curation (equal). **Nobukazu Tanabe:** data curation (equal). **Hiroshi Numao:** data curation (equal). **Hiromasa Ohira:** supervision (equal), validation (equal). **Yoshiyuki Ueno:** supervision (equal). **Atsushi Masamune:** supervision (equal).

## Ethics Statement

This study followed the principles of the Declaration of Helsinki (Fortaleza revision, 2013). Study approval statement: This study was reviewed and approved by the institutional review board of Tohoku University Hospital (approval number: 2021‐1‐377).

## Consent

Due to the retrospective observational study, the institutional review board of Tohoku University Hospital waived the need for written informed consent. The identifying data of the enrolled patients has been delinked, and the authors could not access the individual data.

## Conflicts of Interest

Hidekatsu Kuroda and Hiromasa Ohira are editorial board members of *Hepatology Research*. Yoshiyuki Ueno and Atsushi Hiraoka declare conflicts of interest, and the other authors declare no conflicts of interest for this article.

## Supporting information


**Data S1:** Supporting Information.

## Data Availability

All data generated or analyzed during this study areincluded in this article. Further enquiries can be directed to the corresponding author.
